# One Year of Wastewater Surveillance in South Africa Supporting COVID-19 Clinical Findings Across Two Waves of Infection

**DOI:** 10.3390/microorganisms12112230

**Published:** 2024-11-04

**Authors:** Renée Street, Angela Mathee, Tarylee Reddy, Nomfundo T. Mahlangeni, Noluxabiso Mangwana, Sizwe Nkambule, Candice Webster, Stephanie Dias, Jyoti Rajan Sharma, Pritika Ramharack, Johan Louw, Swastika Surujlal-Naicker, Natacha Berkowitz, Mongezi Mdhluli, Glenda Gray, Christo Muller, Rabia Johnson

**Affiliations:** 1 Environment & Health Research Unit, South African Medical Research Council (SAMRC), Cape Town 7505, South Africa; nomfundo.mahlangeni@mrc.ac.za (N.T.M.); sizwe.nkambule@mrc.ac.za (S.N.); candice.webster@mrc.ac.za (C.W.); 2 Environmental Health Department, Faculty of Health Sciences, University of Johannesburg, Johannesburg 2028, South Africa; angie.mathee@mrc.ac.za; 3 Environment & Health Research Unit, South African Medical Research Council (SAMRC), Johannesburg 2028, South Africa; 4 Biostatistics Unit, South African Medical Research Council (SAMRC), Durban 4091, South Africa; tarylee.reddy@mrc.ac.za; 5 Biomedical Research and Innovation Platform (BRIP), South African Medical Research Council (SAMRC), Tygerberg 7505, South Africa; noluxabiso.mangwana@mrc.ac.za (N.M.); stephanie.dias@mrc.ac.za (S.D.); jyoti.sharma@mrc.ac.za (J.R.S.); pritika.ramharack@mrc.ac.za (P.R.); johan.louw@mrc.ac.za (J.L.); christo.muller@mrc.ac.za (C.M.); rabia.johnson@mrc.ac.za (R.J.); 6 Department of Microbiology, Stellenbosch University, Stellenbosch 7600, South Africa; 7 Centre for Cardio-Metabolic Research in Africa, Division of Medical Physiology, Faculty of Medicine and Health Sciences, Stellenbosch University, Stellenbosch 7600, South Africa; 8 Discipline of Pharmaceutical Sciences, School of Health Sciences, University of KwaZulu-Natal, Westville Campus, Durban 4001, South Africa; 9 Department of Biochemistry and Microbiology, University of Zululand, KwaDlangezwa 3886, South Africa; 10 Scientific Services, Water and Sanitation Department, City of Cape Town Metropolitan Municipality, Cape Town 8000, South Africa; swastika.surujlalnaicker@capetown.gov.za; 11 Community Service and Health, City Health, City of Cape Town, Hertzog Boulevard, Cape Town 8000, South Africa; natacha.berkowitz@capetown.gov.za; 12 Chief Research Operations Office, South African Medical Research Council, Tygerberg 7050, South Africa; mongezi.mdhluli@mrc.ac.za; 13 Office of the President, South African Medical Research Council, Tygerberg 7050, South Africa; glenda.gray@mrc.ac.za

**Keywords:** SARS-CoV-2, COVID-19, viral RNA, wastewater, environmental epidemiology, South Africa

## Abstract

Wastewater-based epidemiology (WBE) has been an important tool for the detection of COVID-19 outbreaks. The retrospective analysis of COVID-19 data is vital to understand the spread and impact of the virus as well as to inform future planning and response efforts. In this study, we evaluated the SARS-CoV-2 RNA levels in wastewater from 21 wastewater treatment plants (WWTPs) in the City of Cape Town (South Africa) over a period of 12 months and compared the (inactive) SARS-CoV-2 viral RNA in wastewater between wave 2 (November 2020 to January 2021) and wave 3 (June 2021 to September 2021). The SARS-CoV-2 RNA expression was quantified in wastewater using quantitative real-time PCR (qRT-PCR) by targeting the nucleocapsid (N) gene, and the resultant signal was normalized to the WWTP design capacity and catchment size. Our findings show that the maximum SARS-CoV-2 RNA signal was significantly higher in wave 3 than in wave 2 (*p* < 0.01). The duration of wave 3 (15 weeks) was longer than that of wave 2 (10 weeks), and the wastewater surveillance data supported the clinical findings, as evidenced by the two distinct waves. Furthermore, the data demonstrated the importance of long-term wastewater surveillance as a key indicator of changing trends.

## 1. Introduction

COVID-19 continues to be a significant global public health issue requiring a multi-pronged response. Wastewater-based epidemiology (WBE) has been used to monitor various pathogens, and the detection of non-infectious RNA fragments of SARS-CoV-2 in wastewater has triggered many countries worldwide to establish wastewater surveillance systems for COVID-19 [1,2,3,4,5]. Such surveillance can provide an early warning for the detection of COVID-19 cases in the community, detect variants, assess trends and track hotspots. This type of surveillance may provide a cost-effective, rapid and reliable source of information on the spread of SARS-CoV-2 in communities. SARS-CoV-2 continues to evolve, and WBE can assist by identifying new potential threats. More than three years into the pandemic, South Africa has experienced four distinct COVID-19 waves (with a fifth wave being very mild) and over 100,000 COVID-19 related deaths [6]. The differences in timing and scale of each wave have been evident at the provincial and district level. Our previous publication indicated that the second wave was driven by the Beta variant; however, the third wave was characterized by the highly transmissible Delta VOC, resulting in the third wave being identified as the Delta wave [7,8]. It is paramount to reflect on lessons learned from continuous wastewater sampling and detail the contribution from different WWTPs. Monitoring the SARS-CoV-2 viral loads in wastewater can assist in recording trends during a high prevalence of infections, while at lower prevalence, it provides an early warning of the (re)emergence of the virus [9]. Therefore, the integration of wastewater data from all COVID-19 waves into the public health response can ensure our preparedness for future pandemics. This study aimed to describe the SARS-CoV-2 RNA signal in wastewater over a 12-month sampling period within the City of Cape Town Metro. The SARS-CoV-2 RNA signal between wave 2 and wave 3 was compared, taking into account the WWTP design capacity and catchment size.

## 2. Materials and Methods

### 2.1. Wastewater Sampling

**Figure 1:**
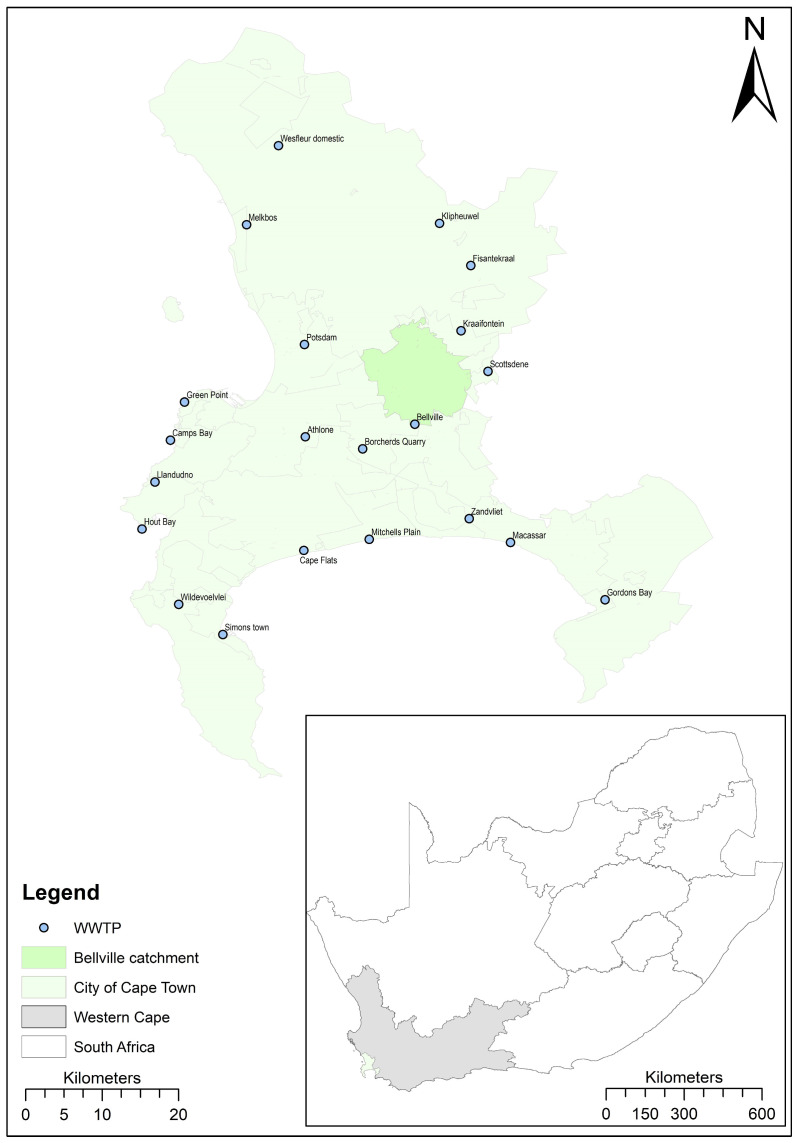
Map of the study sites, highlighting the catchment with the largest fold change (signal) from wave 3 to 2.

Between 2 November 2020 and 31 October 2021, one grab wastewater sample was collected weekly from the influent of 21 of the 24 wastewater treatment plants (WWTPs) in the City of Cape Town over a 52-week (12 month) period (1092 samples in total, Figure 1). Three WWTPs, Wesfleur Industrial, Millers Point and Oudekraal, do not serve residential populations and were excluded from analysis. The wastewater samples were collected using a standardized sampling method as defined in the *SAMRC Wastewater Sampling Guide* (Supp 1). All samples were collected at approximately the same time and on the same day once a week using 500 mL polypropylene autoclavable bottles. Thereafter, the samples were transported to the laboratory on ice for processing.

### 2.2. Sample Concentration and RNA Extraction

RNA extraction was performed by centrifuging 100 mL of an influent sample at 2500× *g* for 20 min. The resultant 2.5 mL pellet was used for total RNA extraction in accordance with the protocol described previously by Johnson et al. [10]. In short, the extracted RNA was homogenized and phase-separated; thereafter, the quality and quantity of total RNA was assessed using nanodrop spectrophotometry before storing the aliquoted RNA (70 µL) at −80 °C.

### 2.3. qRT-PCR Analysis

Extracted RNA was standardized at a concentration of 0.2 µg/µL, and quantitative real-time polymerase chain reaction (qRT-PCR) was performed as previously described by Johnson et al. [11]. The reaction was performed using Bio-Rad iTaq Universal Probes One-Step Kit (Bio-Rad Laboratories, Hercules, CA, USA) in accordance with the manufacturer’s instructions. The presence of SARS-CoV-2 was determined and quantified using the Centers for Disease Control and Prevention (CDC)-approved 2019-nCoV CDC EUA Combined primer/probe kit (Integrated DNA Technologies, Coralville, IA, USA), using the nucleocapsid (N1 and N2) as a target [10]. The cycling conditions were for a one-step reverse transcription and qRT-PCR reaction at 50 °C for 20 min, followed by 95 °C for 3 min, 95 °C for 15 s and 60 °C for 1 min. Standard curves were generated in duplicate by using 10-fold serial dilutions of the 2019-nCoV N Positive Control (Whitehead Scientific, Coralville, IA, USA) (concentration ranged from 200,000 to 2 genome copies (g.c.)/µL). PCR was conducted on an Applied Biosystems™ QuantStudio™ 7 Flex Real-Time PCR System (Thermo Fisher Scientific, Waltham, MA, USA).

### 2.4. Normalization Calculations

Population data for each WWTP catchment area were provided by the City of Cape Town and based on the 2019 population estimates. Although traditionally flow normalization is calculated based on both the catchment population and the daily flow rate, in our context, the daily flow rate data are not readily available. Hence, an alternative approach to normalize the wastewater data was developed to use the design capacity (in mega liters; ML) of each WWTP for the calculations. The design capacity was used to calculate an estimated weekly load of the viral RNA copy number for each WWTP (in viral copy number/day). The population estimates from each WWTP were then used to generate an estimated per capita daily load (genome copies/day/100,000 inhabitants) of the viral RNA markers at the respective WWTPs. In this way, the qRT-PCR quantitative results for SARS-CoV-2 RNA markers were normalized to compensate for the variation in WWTP flow received by each plant, as well as to compensate for the variable population size in each community that is served by the respective WWTPs, for a more accurate comparison between locations. This number was then multiplied by 100 billion [11]. For the remainder of the manuscript, this normalized signal will be referred to as the SARS-CoV-2 RNA signal.

### 2.5. Clinical COVID-19 Case Data

The number of new COVID-19 cases in the City of Cape Town was retrieved from the Western Cape Provincial Government COVID-19 Dashboard [12]. The defined period for wave 2 was from 16 November 2020 to 25 January 2021, with clinical cases peaking between 28 December 2020 and 4 January 2021. The defined period for wave 3 was from 14 June 2021 to 21 September 2021, with clinical cases peaking between 14 August and 20 August.

### 2.6. Statistical Analysis

The SARS-CoV-2 RNA signal was analyzed using descriptive statistics. The correlation between N1 and N2 was assessed using Spearman’s rank correlation. The maximum value observed over the duration of each wave was estimated for each WWTP separately. The Wilcoxon signed-rank test was used to test whether there was a significant difference between the maximum SARS-CoV-2 signal in wave 2 and that in wave 3. Daily case data were smoothed using a 7-day moving average. All analyses were performed using Stata version 15.0 (StataCorp, College Station, TX, USA).

## 3. Results

**Figure 2:**
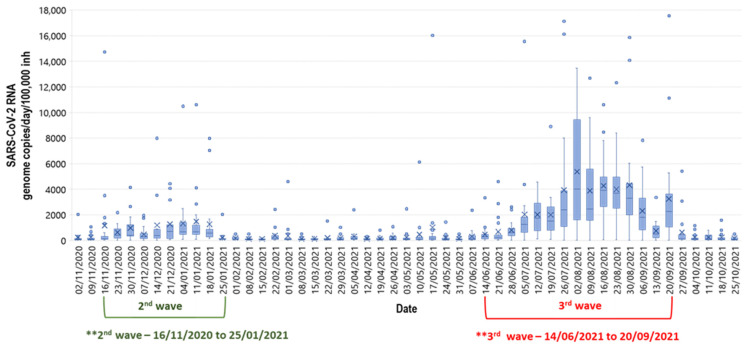
Wastewater surveillance over a 12-month period including two COVID-19 waves. The box plots represent weekly sampling from 21 WWTPs presented in SARS-CoV-2 RNA copies/day/100,000 inhabitants × 100 billion (** normalized by design capacity flow and population).

A total of 1092 samples were collected and analyzed from the 21 WWTPs over 52 weeks (Figure 1). A statistically significant correlation between the N1 and N2 values (Spearman’s rank correlation coefficient, *rho* = 0.91; *p* = 0.0001) was observed. Figure 2 shows the graphical representation across the entire sampling period, which includes wave 2 and wave 3. For illustration purposes, 11 data points were removed to allow for better visual representation (for the graph and box plots only, not in the further analysis). The removed data points are listed in the supplementary data file (Table S1).

**Figure 3:**
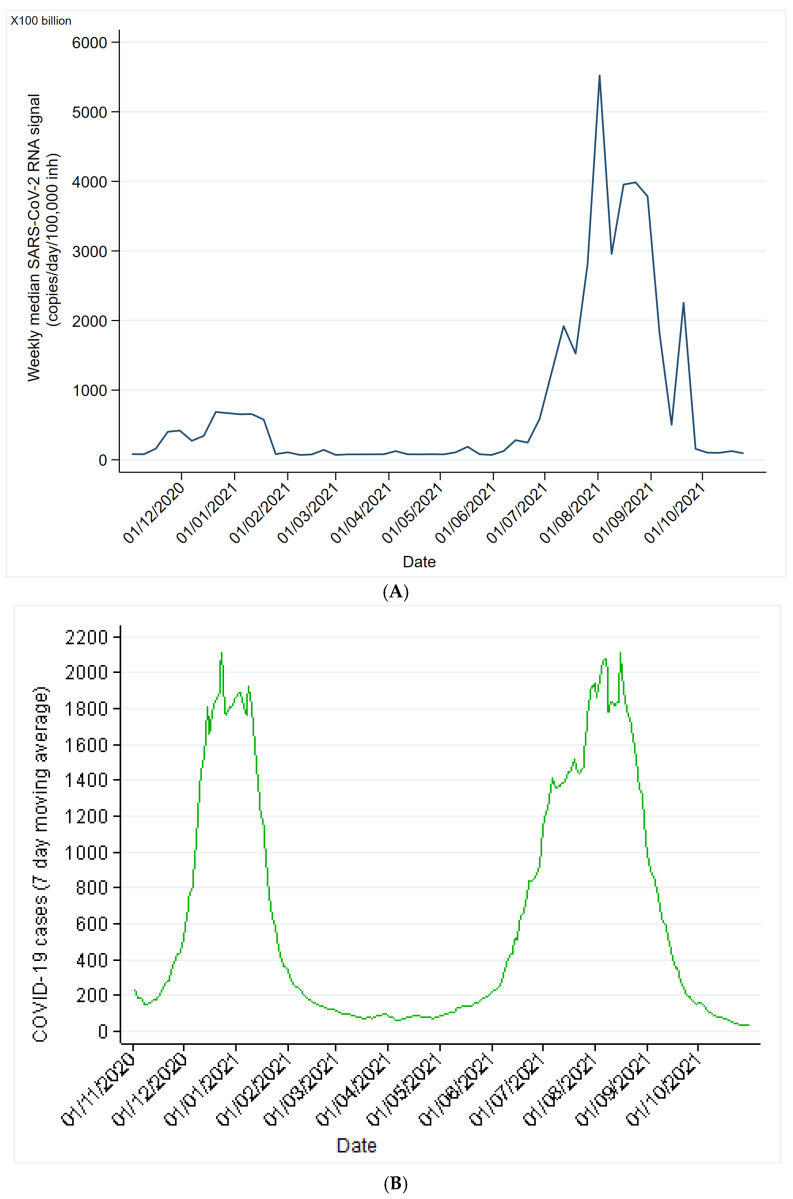
(**A**) Weekly median SARS-CoV-2 signal in wastewater. (**B**) COVID-19 cases over a 12-month period.

**Figure 4:**
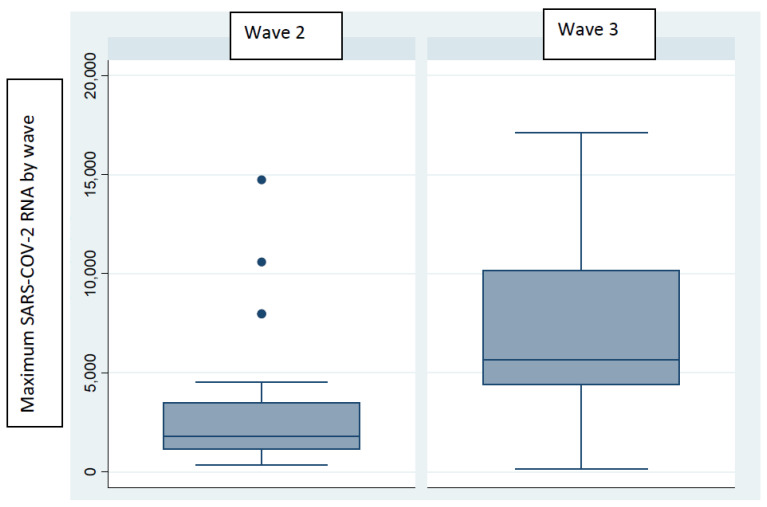
Distribution of the maximum SARS-CoV-2 RNA signal by COVID-19 wave.

Figure 3 shows that the wastewater surveillance data supported the clinical findings, as the two distinct waves occurred over a similar period. The duration of wave 2 was 10 weeks compared with 15 weeks for wave 3. The total number of samples analyzed per wave was 240 and 360 for waves 2 and 3, respectively. The summary median across all the WWTPs during wave 2 was 385 (IQR 158–957) compared with 1798 (IQR 502–3828) in wave 3. Table 1 reveals the maximum signal per WWTP for waves 2 and 3, and Figure 4 shows the distribution of the maximum signal by wave. When comparing the maximum signals per WWTP during wave 2, only three WWTPs (14%) had a higher maximum signal than in wave 3. Bellville WWTP had the highest fold increase at 27.7. Overall, across all the WWTPs, the maximum RNA level in wave 3 was significantly higher than that in wave 2 (*p* < 0.01).

## 4. Discussion

This study was conducted to analyze the SARS-CoV-2 RNA signal observed over a 52-week (12 month) period and the reliability of WBE data, quantifying RNA genome copies from 1092 wastewater samples. This study demonstrated that wastewater surveillance can support clinical data, as the wave and inter-wave periods of the wastewater surveillance method supported the observed clinical cases. An analysis based on WWTP provided deeper insight into the differences in signal distribution over the year. Wastewater surveillance is a less invasive, more inclusive and more feasible method to support clinical surveillance, as it does not require inhabitants to be present for testing individually. Metro-wide sampling proved to be a suitable and efficient surveillance method in a low- or middle-income country (LMIC) setting. The suitability of wastewater surveillance in an LMIC such as South Africa is noteworthy, as there were limited resources, testing kits and human capacity at the onset of the pandemic. Although a targeted screening and testing COVID-19 strategy was adopted, the high testing volumes resulted in the delayed reporting of positive cases. Other characteristics of the COVID-19 virus, such as asymptomatic cases, false negative test results and the high transmissibility of VOCs such as Delta, meant that clinical cases provided skewed results [13].

Our previous research on SARS-CoV-2 variants in wastewater [14] showed that the prevalence of the Beta variant decreased steadily in the Western Cape and that the onset of wave 3 resulted in the Delta variant becoming the predominant variant, driving the third wave. Our wastewater results indicate that the SARS-CoV-2 viral load was higher in wave 3 than in wave 2, while clinical data indicated similar case maximums in both waves (±3000 cases). It is evident from the literature that the Delta variant has a higher viral load than other variants [15]. For instance, in clinical studies, Ong et al. [16] compared the outcomes of patients infected with the Alpha, Beta and Delta variants and observed that the Delta variant increased disease severity. Similarly, Li et al. [17] observed that patients who were infected with the Delta variant had higher viral loads and prolonged viral shedding compared to those infected with the Alpha or Beta variants. The difference in the shedding characteristics of each VOC is important to consider when interpreting wastewater surveillance data. The differential viral shedding does pose a challenge to determining the COVID-19 prevalence using wastewater surveillance [18]. Further research is required to understand the shedding characteristics of different viruses to inform researchers of how to translate the observed results. The results presented in this paper detail the second and third wave of COVID-19 infections in a South African metropolitan area. The wastewater surveillance of SARS-CoV-2 has been shown to be useful and an important approach to track COVID-19 prevalence, complementary to clinical testing [4,5,19,20]. According to a review conducted by Shah et al. [18] on SARS-CoV-2 wastewater surveillance, eight countries from LMICs detected viral RNA in wastewater (Argentina, Brazil, Ecuador, India, Iran, Pakistan, South Africa and Thailand). Arora et al. [21] reported that positive wastewater samples correlated with the surge in COVID-19 cases in India, and Wannigama et al. [22] observed that an increase in SARS-CoV-2 RNA levels in wastewater preceded the rise in COVID-19 cases in Thailand. Other studies also corroborate these findings [23,24,25]. The wastewater surveillance approach allows for the tracking of SARS-CoV-2 in the community among asymptomatic and pre-symptomatic individuals who are not detected during clinical surveillance. These findings confirm the use of WBE to postulate the spread and new resistance outbreaks at a community level.

The global COVID-19 response has been unequal, with some countries and populations bearing the brunt of the pandemic more than others [26]. The wastewater surveillance of SARS-CoV-2 is now widely used, particularly in high-income countries [27,28]. In line with those studies, our results confirm that WBE is possible in an LMIC setting; however, there are certain caveats. South Africa has a comparatively good core of country-wide infrastructure. However, issues have been raised regarding the institutional and financial capability to support both the maintenance and the pressure from expansion. Hence, although WBE may work in certain parts of the country, a nationwide roll out of WBE is unlikely to be effective without some major infrastructural provision. The policies and procedures for clinical COVID-19 testing vary greatly worldwide, and over the past few years, they have been affected by availability, cost and logistical challenges. Currently, wastewater surveillance can support clinical surveillance and guide public health action; however, with more intensive sampling, it is anticipated that it can provide the data necessary to identify outbreaks. In the context of wastewater surveillance, several infrastructural challenges can hinder widespread implementation, particularly in resource-constrained regions. One major issue is the lack of adequate wastewater treatment facilities. Many regions also lack the necessary laboratory capacity for processing samples. Furthermore, inadequate transportation and storage facilities can compromise sample integrity. To overcome these obstacles, future studies should focus on the development of low-cost, portable testing technologies that allow for on-site analysis, thereby reducing the need for extensive laboratory infrastructure. By addressing infrastructural challenges, wastewater surveillance can become a more effective tool for pandemic prevention, preparedness and response, particularly in resource-constrained settings.

## 5. Conclusions

This study evaluated the change in the SARS-CoV-2 RNA signal in wastewater from 21 WWTPs over a 12-month period that included two epidemiological waves of the current ongoing COVID-19 pandemic. This Metro-wide study, taking into account the WWTP design capacity and catchment size, showed the value of temporal wastewater surveillance as a key indicator of changing trends.

## Figures and Tables

**Table 1:** Maximum SARS-CoV-2 RNA copies/day/100,000 inhabitants × 100 billion (normalized by design capacity flow and population) per wastewater treatment plant (WWTP) by COVID-19 wave.

Name of WWTP	Max SARS-CoV-2 Viral Load: Wave 2 (Driven by the Beta Variant)	Max SARS-CoV-2 Viral Load: Wave 3 (Driven by the Delta Variant)	Fold Change (from Wave 3 to 2)
Athlone	2002	10,197	5.1
Borcherds Quarry	14,737	10,845	0.7
Bellville	1126	31,248	27.7
Camps Bay	7969	29,416	3.7
Cape Flats	1224	4441	3.6
Fisantekraal	10,592	83,661	7.9
Gordons Bay	1227	20,298	16.5
Green Point	4524	26,579	5.9
Hout Bay	3157	67,234	21.3
Kraaifontein	1837	17,127	9.3
Klipheuwel	357	162	0.5
Llandudno	3360	15,551	4.6
Melkbosstrand	896	6348	7.1
Macassar	1415	3369	2.4
Mitchells Plain	1325	5549	4.2
Potsdam	747	6026	8.1
Scottsdene	749	4412	5.9
Simons Town	3511	4379	1.2
Wesfleur Domestic	1796	5677	3.2
Wildevoelvlei	7986	5703	0.7
Zandvliet	469	4178	8.9

## Data Availability

Data available upon reasonable request in writing. Additional data is provided in the Supplementary File.

## Supplementary Materials

The following supporting information can be downloaded at: https://www.mdpi.com/article/10.3390/microorganisms12112230/s1, Table S1: Data points removed for visual representation of graphs.
