# Supplementary material for: One Year of Wastewater Surveillance in South Africa Supporting COVID-19 Clinical Findings Across Two Waves of Infection
Source: Microorganisms. 2024 Nov 4;12(11):2230. doi: 10.3390/microorganisms12112230 (PMC11596097; doi:10.3390/microorganisms12112230)
Supplement: Supplementary file 1 [file microorganisms-12-02230-s001.zip › microorganisms-3206920-supplementary.pdf]

Supplementary Files.

Table S1. Data points removed for visual representation of graphs

**Date**	**Wastewater Treatment Plants**	**SARS-CoV-2 RNA copies/day/100 000 inh (x 100 billion)**
12/07/2021	Fisantekraal	27 741
26/07/2021	Camps Bay	29 416
26/07/2021	Green Point	26 579
02/08/2021	Fisantekraal	74 355
02/08/2021	Hout Bay	67 234
09/08/2021	Fisantekraal	28 715
09/08/2021	Gordon's Bay	20 298
16/08/2021	Fisantekraal	33 164
23/08/2021	Fisantekraal	35 212
30/08/2021	Bellville	31 248
30/08/2021	Fisantekraal	83 661
06/09/2021	Fisantekraal	20 197
